# Solid fuel use and early child development disparities in Ghana: analyses by gender and urbanicity

**DOI:** 10.1038/s41370-020-0224-4

**Published:** 2020-05-04

**Authors:** José Ignacio Nazif-Muñoz, John D. Spengler, Raphael E. Arku, Youssef Oulhote

**Affiliations:** 1 Department of Environmental Health–T. H Chan School of Public Health, Harvard University, Boston, MA USA; 2 Faculty of Medicine and Health Sciences, University of Sherbrooke, Longueuil, QC Canada; 3 Department of Environmental Health Sciences, School of Public Health and Health Sciences, University of Massachusetts at Amherst, Amherst, MA USA; 4 Department of Biostatistics and Epidemiology, School of Public Health and Health Sciences, University of Massachusetts at Amherst, Amherst, MA USA

**Keywords:** Solid Fuel use, Early child development, Neurodevelopment, Indoor air pollution, Gender disparities, Ghana

## Abstract

In Ghana, more than 77% of the population depends on biomass fuels for cooking. Previous studies show that solid fuel use (SFU) has adverse effects on pregnancy and child health outcomes. Yet, no previous study considered potential effects on early child development indicators (ECDI), nor how SFU effects may vary by gender, and rural and urban areas. We investigated the associations of SFU with ECDI measures, and whether these associations exhibited sex and urban/rural differences. We used the 2011–2012 Ghana’s Multiple Indicator Cluster Surveys–UNICEF (*N* = 3326 children; 3–4 years). We derived a binary ECDI measure reflecting whether the child is developmentally on track or not from a caregiver-report of ten yes/no/do not know questions designed specifically to assess four domains of early child development: learning-cognition, literacy-numeracy, socio-emotional, and physical. We used multilevel Poisson regressions adjusting for neighborhood, household, mother, and child’s characteristics to estimate covariate-adjusted prevalence ratios (PRs) of the associations between SFU and ECDI and its four dimensions. We run stratified analyses and used *z*-score tests of differences to evaluate effect modification by sex and urbanicity. Overall, 85% of children were exposed to SFU and 28% of children were not developmentally on track. After adjustment for confounders, children exposed to SFU were more likely to be not developmentally on track in comparison with nonexposed children (PR = 1.16; 95% confidence interval, [95% CI]: 1.10,1.22). These associations were stronger in girls (PR = 1.36; 95% CI: 1.03,1.79) in comparison with boys (PR = 0.87; 95% CI: 0.73,1.04). No difference in associations was observed between urban and rural children. Overall, these associations were mainly driven by the literacy-numeracy dimension. In this study, we show that SFU was associated with developmental delays in Ghanaian girls. Policy efforts which tackle SFU should be mindful of gender disparities in susceptibility to indoor pollution.

## Introduction

Globally, 41% of all households rely on solid fuels (wood, charcoal, coal, dung, and crop residues) for cooking and other household needs with consequences for health outcomes in populations [1]. In 2016, household air pollution from cooking with solid fuel contributed to at least 3.6 million cases of premature mortality in low- and middle-income countries (LMICs) [1], making solid fuel use (SFU) a major risk factor for health in LMICs. The disease burden associated with SFU span a range of health outcomes, including respiratory illnesses [2–6], lung cancer [7, 8], and cataracts [9]. Recent evidence also suggested associations with tuberculosis [10], and low birth weight [11]. Due to gender-defined roles in LMICs, women and girls and children who mostly cook and spend significant time near stoves with polluting fuels, are disproportionally affected [12–17].

While the negative impacts of SFU on various health outcomes have been firmly established, only recently the field is shifting to examine how indoor pollution affects early child development (ECD) [18]. We understand ECD as an interactive process, resulting in an ordered progression of perceptual, motor, cognitive, language, socio-emotional, and self-regulation skills [19]. Studies carried out in high-income countries have suggested negative associations between indoor air pollution and ECD. A prospective study in the United States demonstrated that increases in indoor air pollutants from fossil fuel burning were associated with decreases in information processing speed and augmented symptoms of attention-deficit/hyperactivity disorder in children aged 7 to 9 years [20]. A study in Spain examined the impact of indoor air pollution from gas cooking and found a negative association with mental development in children (aged 11–22 months) [21].

Cross-national research in LMICs has recently suggested the impact of various social and demographic factors on ECD such as, children’s age, breastfeeding, household characteristics, and marital status of the mothers [15, 22, 23]. Nevertheless, it has been largely recognized that our understanding of the impact of indoor pollution on ECD is very limited in LMICs, where SFU is prevalent [19]. With the sole exception of one study carried out in Ghana, which observed negative effects of cooking fuel type (firewood, charcoal, and kerosene) on school attendance in children aged 6–18 years [24], no other study has assessed the effects of SFU on children’s developmental status.

In this article, we used Ghana’s Multiple Indicator Cluster Survey (MICS) data to investigate whether SFU is associated with ECD among children, and if the association varies by sex, and rural–urban residence among children. To better understand the different implications of SFU, we additionally evaluated its impact on four developmental domains: learning-cognition, literacy-numeracy, physical development, and socio-emotional development.

## Methods

### Study population and data sources

We used cross-sectional MICS data of Ghana, collected between 2011 and 2012. Briefly, the MICS is a cross-sectional nationally representative household survey that collects health information through a face-to-face interview. This survey has covered 116 countries, many over several rounds of surveys producing trend data and therefore analyses over time and across and within countries is feasible [25]. A two-stage cluster sampling design is employed to sample the households before collecting information on women (aged 15–49 years), men (aged 15–49), and their children (0–5 years). 3326 of children (3–5 years) in Ghana that were subject of this study.

Detailed information of the survey is available at http://mics.unicef.org/ and a complete overview is provided by Khan and Hancioglu [25].

### Early child development indicators

The MICS included an Early Child Development Index (ECDI) as a measure of the developmental status of children aged 36 to 59 months within four domains: literacy-numeracy, physical, social-emotional development, and learning [26]. This index measures the developmental potential of the child through culture-free instruments based on real-time observation. Early child developmental outcomes are inferred from a 10-item questionnaire (Table 1), and were categorized as child “developmentally on track/not on track” in the four domains and in general development as follows:

- Literacy-numeracy: Children are developmentally on track if they can do at least two of the following: identify/name at least ten letters of the alphabet; read at least four simple, popular words; and/or know the name and recognize the symbols of all numbers from 1 to 10.
- Physical development: Child is on track If she/he can pick up a small object with two fingers, like a stick or rock from the ground, and the mother/primary caregiver does not indicate that the child is sometimes too sick to play,
- Social-emotional: The child is on track if two of the following are true: the child gets along well with other children; the child does not kick, bite or hit other children; and the child does not get distracted easily.
- Learning: If the child follows simple directions on how to do something correctly and/or when given something to do, and is able to do it independently, then the child is considered to be developmentally on track in the learning domain.
- General development: Children are identified as being developmentally on track if they are on track in at least three of the four domains.

**Table 1:** Dimensions and questions of the Early Child Development Index (ECDI).

Dimension	Questions
(i) Literacy-numeracy	• Can identify or name at least ten letters of the alphabet? • Can read at least four simple, popular words? • Does know the name and recognize the symbol of all numbers from 1 to 10?
(ii) Learning-cognition	• Does follow simple directions on how to do something correctly? • When given something to do, is able to do it independently?
(iii) Physical development	• Can pick up small object with two fingers, like a stick or a rock from the ground • Is sometimes too sick to play?
(iv) Social-emotional development	• Does get along well with other children? • Does kick, bite, or hit other children or adults? • Does get distracted easily?

A child who answered “yes” to at least three of the four domains with a minimum two “yeses” for each domain is considered as either “being developmentally on track” or “having no developmental delays”. As a robustness check we also used a standardized ECDI score and results were similar in magnitude and direction to the binary measure. (Results are presented in Appendix in Table A3). Analyses of each of the four domains were also established following the aforementioned classification.

A validation study for the ECDI was conducted in Jordan and the Philippines among 1800 children evenly distributed by sex. Urban and rural regions were represented, and a subsample of children was used to test reliability. The recommended set of questions was also tested alongside several other validated tools, including the Early Development Instrument and the Strengths and Difficulties Questionnaire. Factor analyses demonstrated excellent reliability and validity of the questionnaire [26].

### Solid fuel use

Our key exposure variable of interest is SFU, an indicator for household air pollution. This variable was dichotomized into exposed vs. nonexposed. Children living in households/homes that reported using coal, charcoal, wood, straw/shrubs, animal dung, or agricultural crop residue were defined as ‘exposed’, whereas ‘nonexposed’ children were those habiting households that reported using electricity, liquefied petroleum gas, natural gas, and/or biogas.

### Covariates and potential confounders

The following variables related to child, mother, and household characteristics that are associated with ECD [16, 22] were chosen a priori as covariates in the models:

#### Children’s characteristics

Sex, age (in months); vitamin supplementation (never/ever); stunting status (no/yes); breastfeeding (never/ever); and attending early child education (no/yes).

#### Mother’s characteristics

Age (in years); education (none and preschool; primary and secondary/tertiary and more studies); and marital status (married/not married).

#### Household’s characteristics

Wealth index score (this index incorporates household characteristics (i.e., electricity, water facilities, number of rooms, type of toilet, and building material of roofs, floors, walls), presence of material goods (i.e., television, telephone, refrigerator) and ownership of a computer, camera, and bank account, among other goods. The original index provided by UNICEF included the type of fuel used in the home, so we reconstructed a new index excluding this characteristic to avoid overadjustment in our models. The index was therefore categorized into quintiles with the highest quintile reflecting the wealthiest households and the lowest one reflecting the poorest.

### Statistical analysis

We describe the prevalence of SFU and children’s developmental status (i.e., children not developmentally on track) and the distribution of each of the covariates. Depending on the covariate, we used *t* tests or analysis of variance to examine univariate associations between ECDI and participants’ characteristics.

To study the associations between SFU, ECDI, and each subdomain, we used multilevel Poisson regression models with robust variance to take into account the multistage probability sampling design with sampling within neighborhoods [27]. We also considered the survey weights to provide estimates that are generalizable to the entire 3–5 years children population. Poisson regressions are more appropriate and provide prevalence ratios (PRs) that are preferable to odds ratios from logistic regression which overestimate relative risks when the outcome is common. This approach provides correct estimates of the PRs and their variances in cross-sectional studies [28]. For robustness check we also applied Logistic regression models and results are consistent with the Poisson models. (Results are available in Appendix in Table A4).

In additional analyses, we explored whether the association between SFU and ECDI and its subdomains differed by gender or urbanicity status. Differences in the associations in sex (boys and girls) or urbanicity (rural and urban) groups were tested by comparing the value of *d*/SE_*d*_ to the standard normal distribution, where *d* is the difference between the two estimates, and $SE_d = \sqrt {SE_1^2 + SE_2^2}$ is the standard error of the difference [29]. (This is based on a height-for-age of <2 standard deviations below the WHO standard).

## Results

### Children’s, mothers’, and household characteristics

The number of subjects and the distribution of the exposure, outcomes, and other important characteristics are presented in Table 2. Information from 3325 children aged 3–5 years was used in this analysis. In this sample, 85% of households used solid fuels as primary source of energy for cooking. Overall, 51% of the children were males, more than 50% were 3 years of age, almost all were breastfed (99%), 59% attended an early educational program, 17% received intake of vitamin, 18% present stunting, and 49% lived in urban settings. Mothers’ characteristics indicate that 93% were married, 49% of the mother’s population were between 25 and 35 years of age, and more than 50% had less or equal than primary education. Lastly, 28% of children were not developmentally on track on the global ECDI with, respectively, 69%, 27%, 11%, and 2% being not developmentally on track in literacy-numeracy, socio-emotional development, learning-cognition, and physical development.

**Table 2:** Population characteristics.

Variable	*N*	(%)
Sex		
Males	1650	49.6%
Females	1676	50.4%
Age		
3 years age	1688	51.9%
4 years age	1637	48.1%
Vitamin supplementation		
Yes	542	16.7%
No	2783	83.7%
Stunting		
Yes	914	29.0%
No	2412	71.0%
Breastfed		
Yes	3208	98.7%
No	118	1.3%
Attended early education program		
Yes	1907	58.7%
No	1419	41.3%
Mother’s age		
15–24	733	24.0
25–35	1512	49.7
35–49	801	26.3
Mother’s education		
Preschool	1826	54.9%
Primary	570	17.1%
Secondary	930	27.6%
Marital status		
Married	2666	92.8%
Not married	660	7.2%
Urbanicity		
Urban	1070	32.1%
Rural	2256	67.9%
Wealth		
Quintile 1 (poorest)	1162	35.8%
Quintile 2	746	23.0%
Quintile 3	483	14.9%
Quintile 4	458	14.1%
Quintile 5 (affluent)	391	12.1%
Children not developmentally on track:		
Global	845	26.3%
Children developmentally on track:		
Global	2481	73.7%
Children not developmentally on track:		
Literacy-numeracy	2437	75.3%
Children developmentally on track:		
Literacy-numeracy	889	24.7%
Children not developmentally on track:		
Learning-cognition	357	11.8%
Children developmentally on track		
Learning-cognition	2969	88.2%
Children not developmentally on track		
Physical development	72	2.2%
Children developmentally on track		
Physical development	3254	97.8%
Children not developmentally on track		
Socio-emotional development	795	24.7%
Children developmentally on track		
Socio-emotional development	2531	75.3%
Total	3326	

Table 3 presents univariate associations between ECDI and the participants’ characteristics. We observe that younger children, girls, children not attending an early education program, not having taken vitamin supplementation, and being stunted were more likely to be not developmentally on track. Regarding mothers’ characteristics, children of mothers with lower education and not married were more likely to be not developmentally on track. Finally, children from urban areas and in the lower wealth quintile were more likely to be not developmentally on track.

**Table 3:** Univariate associations between EDCI and population characteristics.

	Early child development binary score		
	*N* (%)	Prevalence ‘not on track’	*p* value
Children’s characteristics			
Sex			<0.0001
Male	1650 (49.6)	21%	
Female	1676 (50.4)	28%	
Age (years)			<0.0001
3	1688 (51.9)	30%	
4	1637 (48.1)	19%	
Vitamin			0.223
Yes	2704 (83.3)	25%	
No	542 (16.7)	22%	
Stunting			<0.0001
Yes	914 (29.0)	34%	
No	2412 (70.9)	22%	
Breastfed			0.118
Yes	3208 (98.8)	25%	
No	118 (1.2)	13%	
Attending early education program			<0.0001
Yes	1907 (58.7)	19%	
No	1419 (41.3)	37%	
Mothers’ characteristics			
Mother’s age			0.274
15–24	733 (24.1)	27%	
25–35	1512 (49.7)	22%	
36–49	801 (26.3)	25%	
Mother’s education			<0.0001
None and preschool	1826 (55.0)	31%	
Primary and secondary	570 (17.1)	24%	
Tertiary and more	930 (27.9)	20%	
Marital status			0.054
Married	2665 (92.8)	23%	
Not married	660 (7.2)	31%	
Household characteristics			
Wealth			<0.0001
Quintile 1	1161 (35.8)	32%	
Quintile 2	746 (23.0)	27%	
Quintile 3	483 (14.9)	23%	
Quintile 4	458 (14.1)	26%	
Quintile 5	391 (12.1)	15%	
Urbanicity			0.001
Rural	2256 (67.8)	28%	
Urban	1070 (32.1)	21%	
Exposure of interest			
Solid fuel use			<0.0001
Yes	3040 (91.4)	27%	
No	286 (8.6)	14%	

*n* = 3326; unweighted.

In Table A1 (Supplementary material), we describe univariate analysis between each ECDI subdomain and participants’ characteristics. All children, mother, and household characteristics, except breastfeeding and marital status, were associated with literacy-numeracy development. Regarding learning-condition, only stunting, attending early child education, and maternal education were associated with this subdomain. With reference to physical development, only attending early child education was significantly associated with this domain. Finally, socio-emotional development domain was associated with sex, age, stunting, breastfeeding, maternal age, attending early child education, and urbanicity.

### Associations between SFU and ECDI and its subdomains

In univariate associations, SFU was associated with lower developmental status. Children exposed to SFU were 1.82 (95% CI: 1.77, 1.88) more likely to not be developmentally on track. A similar finding was observed for literacy-numeracy (PR = 1.84; 95% CI: 1.73, 1.95), learning-cognition (PR = 1.53; 95% CI: 1.48, 1.59) and physical development (PR = 4.37; 95% CI: 2.94, 6.51).

**Fig. 1:**
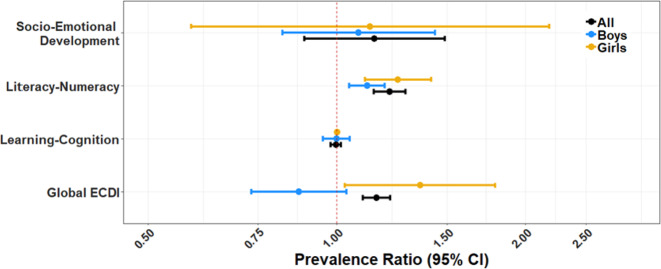
Associations between SFU and ECDI and its subdomains stratified by sex. Models were adjusted for the following variables: child’s age, breastfeeding, attending early education program, vitamin supplementation, stunting, mother’s age, mother’s education, mother’s marital status, urbanicity, and wealth index of the household.

**Fig. 2:**
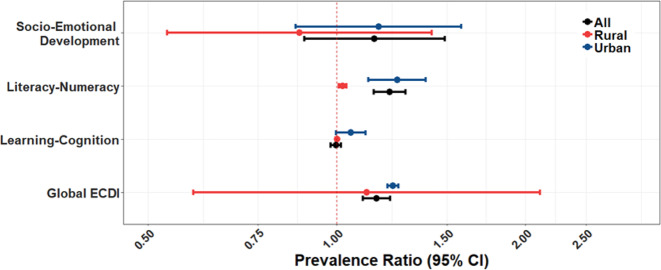
Associations between SFU and ECDI and its subdomains stratified by urban/rural status. Models were adjusted for the following variables: child’s sex, child’s age, breastfeeding, attending early education program, vitamin supplementation, stunting, mother’s age, mother’s education, mother’s marital status, and wealth index of the household.

Figures 1 and 2 show PRs for the multivariable analyses adjusting for potential confounders. Children exposed to SFU were 16% (PR = 1.16; 95% CI: 1.10, 1.22) more likely to be not developmentally on track. Regarding the four subdomains, children exposed to SFU were more likely to be not developmentally on track in the literacy-numeracy (PR = 1.22 (95% CI: 1.15, 1.29) domain. No association was observed for socio-emotional (PR = 1.15; 95% CI: 0.89, 1.49) and learning-cognition (PR = 1.00; 95% CI: 0.98, 1.02) domains. We do not report associations with the physical domain since the models did not converge given the low number of children not being developmentally on track in this dimension. For this we attempted both Poisson and logistic regression models.

In analyses investigating effect modification by gender (Fig. 1), we observed that SFU mainly affected girls. Girls exposed to SFU were 36% (95% CI: 1.03; 1.79) more likely to be developmentally delayed in comparison with girls not exposed to SFU. No such association was observed in boys (PR = 0.87 95% CI: 0.73, 1.04). Analyses of the differences between these two estimates showed that they were significantly different (*p*-_difference_ = 0.008) (all results corresponding to differences are in Table A2). Regarding ECDI developmental subdomains, SFU was significantly associated with literacy-numeracy development in both girls (PR = 1.25; 95% CI: 0.11; 0.41) and boys (PR = 1.12; 95% CI: 0.05; 0.19) with a p-difference approaching significance (*p*-_difference_ = 0.11). No other associations were observed with other ECDI subdomains.

In analyses investigating effect modification by urbanicity status (Fig. 2), we did not observe any significant differences in the associations between SFU and ECDI (*p*-_difference_ = 0.77), although the association was only significant in urban children (PR = 1.23; 95% CI: 1.21; 1.25), compared with rural children (PR = 1.12; 95% CI: 0.59; 2.11). Results per domains, however, show different patterns, in literacy-numeracy we observe a stronger association in urban children (PR = 1.25; 95% CI: 1.12, 1.39) compared with rural children (PR = 1.02; 95% CI: 1.01, 1.04). Our difference analyses suggest that SFU affects more strongly the literacy-numeracy domain in urban children than in rural children (*p*-_difference_ = 0.0002). In learning we observe a stronger association in rural children (PR = 1.05; 95% CI: 1.03, 1.08) compared with urban children (PR = 0.97; 95% CI: 0.94, 1.01). Our difference analyses suggest that SFU affects more strongly the learning domain in rural children than in urban children (*p*-difference = 0.001). We did not observe differences between urban and rural children for socio-emotional development.

## Discussion

SFU has been considered a proxy indicator of household air pollution exposure in several global analyses in LMICs (2–4,6). We used a nationally representative sample of Ghanaian children to examine the associations between household’s SFU for cooking and ECD indicators, and whether the association varies across boys and girls, and rural and urban areas. Our study shows that SFU is independently and positively associated with delays in ECDI in Ghana, particularly in girls. We also observed that several covariates such as attending an early education program, or mother’s education were also associated with children’s developmental status.

Our results regarding SFU and ECDI in Ghana suggest that burning solid fuels may delay children’s early development. The literature points out that children’s brain maturation [30] and their central nervous system are significantly and negatively affected when high levels of carbon monoxide, hydrogen cyanide, ammonia, nitrogen oxide among other compounds, are liberated [31]. Further, children are particularly vulnerable to household pollution since, relative to adults, they have a higher breathing rate to body size ratio [32]. It is also noteworthy that the effects of SFU on the brain at that early ages can be life lasting. SFU thus may condition children’s capacity to learn over time affecting also their productivity. This ultimately has important economic implications for both individuals and society broadly.

In terms of sex differences, our results suggest that exposure to SFU follows a gendered inequality path. In Ghana, girls exposed to SFU were 36% more likely to exhibit developmental delays. Girls may have more frequent exposure to pollutants from cooking fuels than boys, since they spend more time near fires and particularly in the kitchen [33], therefore exposed to higher levels of indoor air pollutants. While other studies [14, 34] have pointed that exposure to SFU disproportionally affects adult women’s health and education attainment relative to men’s, we, on the other hand, suggest that this difference may commence at earlier stages of life, and thus its lasting consequences in domains such as literacy-numeracy should be studied further. Lastly, since cooking activities have also considerable cultural effects on developing and strengthening both families and communities [35, 36], it is important to carry out more research which explains how alternatives such as clean cooking practices could be integrated in households which rely on SFU.

For urbanicity, we notice that when we stratified this variable, urban children exposed to SFU are more likely to show delays in ECDI and its subdomain of literacy-numeracy relative to children no exposed to SFU. This association was weaker in the case of rural children. It is worth mentioning that the results from stratified analyses in rural areas should be interpreted with caution. Given the very high prevalence of SFU in rural areas (<3%), very few children in these areas were not exposed to SFU, therefore yielding potential issues of data scarcity. Nevertheless, the presence of a stronger negative association in urban children, especially for the general ECDI, may be partially explained by the fact that in Ghana outdoor air pollution in urban territories is also higher in comparison with rural areas [37, 38], probably potentiating the exposure from indoor pollutants, which has been consistently shown to affect children’s development [32]. In short, one potential explanation that researchers should explore further is to study the extent under which the presence of indoor pollution outweighs quality of outdoor air in urban territories.

The association between SFU and ECDI was strongly attenuated after other factors were considered, hence at least two covariates, introduced in our analyses, merit some discussion: attending an early education program and mother’s education. We observed that attending early education was associated with ECDI and in each of the four domains. Indeed, activities carried out in formal educational settings seem to be excellent vehicles to stimulate and strengthen children’s development. Nevertheless, while these activities may indeed foster development, it could also be the case that children attending these settings are also less exposed to SFU since they are not at the house. Secondly, the literature usually recognizes mother’s education to be associated with child development by capturing differences in access to resources such as housing, cognitively stimulating materials and experiences, and maternal expectations and styles, among others [39]. In this study, we cannot discard that mother’s education could also be related with specific cooking practices and habits such as housing ventilation, limiting the presence of children in cooking areas and/or decisions over what type of fuel to use within the household when they have the possibility to afford better ones (for instance switching from wood to charcoal). In this regard, more research is needed to investigate whether mother’s education—as well as father’s—in Ghana are also associated with preventive practices aim to explicitly reduce children’s exposure to indoor toxic pollutants.

Our interpretations must be tempered. First, while our main indicator is based on the main type of SFU in each household, many households rely on more than one type of fuel, specially for cooking and heating. Indeed, more research is needed to study the effects of animal dung or agricultural crop residue relative to wood or charcoal since the former is much more inefficient than the latter, but both are extremely toxic [40]. In this regard it would be also extremely beneficial if MICS would consider the introduction of devices which help to properly measure indoor pollution, since currently this survey only provides information of SFU as a proxy for the latter. It is also important to consider that in Ghana other sources of household pollution co-exist. For instance, mosquito coils are usually burnt, and therefore these could also add contaminants to the environment where children play [41]. In this regard pediatric air pollution research requires extensive collaborations, which devise precise and efficient strategies on how to better monitor child development variation in relation to indoor pollution. Simultaneously, it is also important to investigate children’s outdoor playing time since this may also limit the effects of indoor pollution areas relatively free of external contamination.

Last, the observational nature of the data may not allow us to identify with more precision how SFU directly affects ECD. While confounding can never be completely ruled out, our findings of SFU hold after considering other control variables identified in the literature. It is important to consider that our most important variable is a twofold proxy for indoor pollution and cooking practices. However, it has been shown that estimates of the prevalence of household SFU from probability sampling surveys can be made with more confidence than estimates of actual pollutant exposures [42]. Indeed, the fact that existing epidemiological studies of SFU and related health outcomes have achieved reasonably consistent results with binary exposures indicates that this exposure measure may prove to be valuable for environmental epidemiology studies. Moreover, the lack of studies investigating the effects of SFU on child neurodevelopment in LMICs makes this study valuable, and a starting point to further investigations with a better assessment of exposure to indoor air pollutants including information on stove type, cooking/heating practices, and time-activity patterns.

## Conclusion

Our study provides novel findings on the association between SFU and ECD. The study has important implications for exploring further the design and impact of different policies [25]. In tandem with the Sustainable Development Goals (SDG) government policies directed to improve children’s development, which is established in SDG 4 ‘Quality Education,’ particularly its target 4.2, should continue examining the expansion of early education programs by promoting women’s education as it is established in target 4.1. Simultaneously, governments should investigate how incentives and subsidies can increase the use of clean sources of energy instead of solid fuel in households, as both SDG 3 ‘Good Health and Well-Being’ target 3.9 and SDG 7 ‘Affordable and Clean Energy’ target 7.1 explicitly recognize to be necessary steps to curb down indoor pollution [43]. Lastly, more research is needed to assess whether in Ghana significant contributors to household energy change use have been introduced in the past decade to determine what types of government interventions in the context of environmental policies may be mitigating delays in children’s development indicators.

## Supplementary information

Supplementary Appendix

## Data Availability

All analyses were carried out with Stata 14 [44]. Stata codes are available upon request.
