# Supplementary material for: Solid fuel use and early child development disparities in Ghana: analyses by gender and urbanicity
Source: J Expo Sci Environ Epidemiol. 2020 May 4;30(4):698–706. doi: 10.1038/s41370-020-0224-4 (PMC8075970; doi:10.1038/s41370-020-0224-4)
Supplement: Supplementary file 1 — Supplementary Appendix [file 41370_2020_224_MOESM1_ESM.docx]

**Appendix**

**Table A1** Univariate associations between early child development index subdomains, and participants’ characteristics (n=3,325; unweighted)

|  | **N (%)** | **Literacy-Numeracy** | | **Learning-cognition** | | **Physical** | | **Socio-emotional** | |
| --- | --- | --- | --- | --- | --- | --- | --- | --- | --- |
|  |  | **Prevalence ‘not on track’** | ***p*-Value** | **Prevalence ‘not on track’** | ***p*-Value** | **Prevalence ‘not on track’** | ***p*-Value** | **Prevalence ‘not on track’** | ***p*-Value** |
| ***Children’s characteristics*** |  |  |  |  |  |  |  |  |  |
| Sex |  |  | 0.018 |  | 0.4 |  | 0.495 |  | 0.001 |
| *Male* | 1676 (50.4) | 71% |  | 10% |  | 3% |  | 30% |  |
| *Female* | 1649 (49.6) | 66% |  | 11% |  | 2% |  | 22% |  |
| Age (years) |  |  | <0.0001 |  | 0.119 |  | 0.137 |  | 0.06 |
| *3* | 1687 (51.9) | 79% |  | 12% |  | 3% |  | 29% |  |
| *4* | 1562(48.1) | 51% |  | 10% |  | 2% |  | 24% |  |
| Vitamin |  |  | 0.001 |  | 0.247 |  | 0.152 |  | 0.153 |
| *Yes* | 2703 (83.3) | 70% |  | 11% |  | 1% |  | 26% |  |
| *No* | 542 (16.7) | 60% |  | 9% |  | 2% |  | 30% |  |
| Stunting |  |  | <0.0001 |  | 0.031 |  | 0.127 |  | 0.002 |
| *Yes* | 914 (29.1) | 83% |  | 14% |  | 2% |  | 31% |  |
| *No* | 2235 (71.0) | 63% |  | 9% |  | 1% |  | 25% |  |
| Breastfed ^a^ |  |  | 0.257 |  | 0.112 |  | N/A^a^ |  | 0.053 |
| *Yes* | 3207 (98.8) | 69% |  | 10% |  | 100% |  | 26% |  |
| *No* | 40 (1.2) | 58% |  | 22% |  | 0% |  | 9% |  |
| Attending early education programme |  |  | <0.0001 |  | 0.01 |  | 0.041 |  | 0.024 |
| *Yes* | 1905 (58.7) | 96% |  | 9% |  | 9% |  | 24% |  |
| *No* | 1340 (41.3) | 56% |  | 14% |  | 13% |  | 30% |  |
| ***Mothers’ characteristics*** |  |  |  |  |  |  |  |  |  |
| Mother’s age |  |  | 0.016 |  | 0.585 |  | 0.514 |  | 0.07 |
| *Mother’s age 15-24* | 733 (24.0) | 71% |  | 10% |  | 2% |  | 30% |  |
| *Mother’s age 25-35* | 1513 (49.7) | 65% |  | 10% |  | 3% |  | 24% |  |
| *Mother’s age 36-49* | 800 (26.3) | 72% |  | 11% |  | 2% |  | 27% |  |
| Mother’s education |  |  | <0.0001 |  | <0.070 |  | 0.803 |  | 0.579 |
| *None and pre-school* | 1826 (55.0) | 86% |  | 13% |  | 3% |  | 25% |  |
| *Primary and secondary education* | 570 (17.1) | 73% |  | 9% |  | 2% |  | 29% |  |
| *Tertiary education and more* | 929 (27.9) | 53% |  | 10% |  | 2% |  | 26% |  |
| Marital status |  |  | 0.742 |  | 0.39 |  | 0.242 |  | 0.159 |
| *Married* | 2664 (92.8) | 68% |  | 10% |  | 2% |  | 25% |  |
| *Not married* | 208 (7.2) | 69% |  | 13% |  | 3% |  | 31% |  |
| ***Household Characteristics*** |  |  |  |  |  |  |  |  |  |
| Wealth |  |  | <0.0001 |  | 0.721 |  | 0.515 |  | 0.507 |
| *Wealth quintile 1* | 1161 (35.9) | 89% |  | 11% |  | 3% |  | 26% |  |
| *Wealth quintile 2* | 746 (23.0) | 83% |  | 11% |  | 2% |  | 24% |  |
| *Wealth quintile 3* | 482 (14.9) | 66% |  | 10% |  | 2% |  | 26% |  |
| *Wealth quintile 4* | 458 (14.1) | 66% |  | 13% |  | 3% |  | 30% |  |
| *Wealth quintile 5* | 391 (12.1) | 43% |  | 9% |  | 1% |  | 26% |  |
| Urbanicity |  |  | <0.0001 |  | 0.398 |  | 0.154 |  | 0.068 |
| *Urban* | 1069 (32.1) | 55% |  | 10% |  | 2% |  | 10% |  |
| *Rural* | 2256 (67.9) | 80% |  | 11% |  | 3% |  | 11% |  |
| **Variables of interest** |  |  |  |  |  |  |  |  |  |
| Solid fuel use |  |  | <0.0001 |  | <0.0001 |  | <0.0001 |  | 0.293 |
| *Yes* | 3039 (91.4) | 74% |  | 11% |  | 3% |  | 27% |  |
| *No* | 286 (8.6) | 38% |  | 7% |  | 1% |  | 25% |  |

^a^ No children were not breastfed and were not on track in the physical dimension, therefore it was not possible to assess the association between these two variables.

**Table A2 Z**-score tests of differences to evaluate effect modification by sex and urbanicity in ECDI, Literacy-numeracy, Learning and Socio-emotional development

|  | **Coefficient** | | **SE** | | **d** | **SE (d)** | **Z-score** |
| --- | --- | --- | --- | --- | --- | --- | --- |
| Sex | Boys | Girls | Boys | Girls |  |  |  |
| *ECDI* | -0.14 | 0.31 | 0.09 | 0.14 | -0.45 | 0.17 | 2.67 |
| *Literacy numeracy* | 0.23 | 0.11 | 0.06 | 0.03 | 0.11 | 0.07 | 1.61 |
| *Learning Sex* | 0.00 | 0.00 | 0.03 | 0.00 | 0.00 | 0.03 | 0.08 |
| *Socio-emotional development* | 0.08 | 0.12 | 0.14 | 0.34 | -0.04 | 0.36 | 0.12 |
| Urbanicity | Urban | Rural | Urban | Rural |  |  |  |
| *ECDI* | 0.21 | 0.11 | 0.01 | 0.32 | -0.10 | 0.32 | 0.30 |
| *Literacy numeracy* | 0.22 | 0.02 | 0.01 | 0.05 | -0.20 | 0.05 | 3.66 |
| *Learning* | 0.03 | 0.05 | 0.02 | 0.01 | 0.08 | 0.02 | 3.33 |
| *Socio-emotional development* | 0.15 | -0.14 | 0.16 | 0.25 | -0.29 | 0.29 | 0.99 |

**Table A3** Associations between SFU and ECDI standardized score. Mixed effect regression models were adjusted for the following variables: child’s age, breastfeeding, attending early education program, vitamin supplementation, stunting, mother’s age, mother’s education, mother’s marital status, urbanicity, and wealth index of the household.

|  | **Coefficient** | **95% Confidence intervals** | |
| --- | --- | --- | --- |
| *All children* | -0.177 | -0.357 | 0.000 |
| *Boys* | 0.107 | -.0105 | 0.226 |
| *Girls* | -0.227 | -0.348 | -0.106 |
| *Rural children* | -0.034 | -0.07 | -0.007 |
| *Urban children* | -0.164 | -0.39 | 0.005 |

**Table A4** Associations between SFU and ECDI and its subdomains. Logistic models were adjusted for the following variables: child’s age, breastfeeding, attending early education program, vitamin supplementation, stunting, mother’s age, mother’s education, mother’s marital status, urbanicity, and wealth index of the household.

| **All children** | **Odds ratio** | **95% Confidence intervals** | |  |
| --- | --- | --- | --- | --- |
| *ECDI* | 1.250 | 1.223 | 1.277 | |
| *Literacy numeracy* | 1.522 | 1.160 | 1.995 | |
| *Learning Sex* | 1.005 | 0.738 | 1.370 | |
| *Socio-emotional development* | 1.178 | 0.742 | 1.871 | |
| **Boys** |  |  |  | |
| *ECDI* | 0.622 | 0.384 | 1.008 | |
| *Literacy numeracy* | 1.040 | 1.000 | 1.090 | |
| *Learning Sex* | 1.109 | 0.967 | 1.271 | |
| *Socio-emotional development* | 0.927 | 0.526 | 1.635 | |
| **Girls** |  |  |  | |
| *ECDI* | 1.638 | 0.907 | 2.958 | |
| *Literacy numeracy* | 1.978 | 1.020 | 3.837 | |
| *Learning* | 1.746 | 0.852 | 3.578 | |
| *Socio-emotional development* | 1.191 | 0.296 | 4.797 | |
| **Rural** |  |  |  | |
| *ECDI* | 1.169 | 0.625 | 2.185 | |
| *Literacy numeracy* | 1.525 | 1.269 | 1.832 | |
| *Learning* | 0.534 | 0.208 | 1.370 | |
| *Socio-emotional development* | 1.207 | 0.632 | 2.302 | |
| **Urban** |  |  |  | |
| *ECDI* | 1.895 | 1.219 | 2.945 | |
| *Literacy numeracy* | 1.855 | 1.487 | 2.315 | |
| *Learning* | 2.018 | 1.304 | 3.124 | |
| *Socio-emotional development* | 0.797 | 0.355 | 1.788 | |
